# Polarization domain wall pulses in a microfiber-based topological insulator fiber laser

**DOI:** 10.1038/srep29128

**Published:** 2016-07-06

**Authors:** Jingmin Liu, Xingliang Li, Shumin Zhang, Han Zhang, Peiguang Yan, Mengmeng Han, Zhaoguang Pang, Zhenjun Yang

**Affiliations:** 1 College of Physics Science and Information Engineering, Hebei Advanced Thin Films Laboratory, Hebei Normal University, Shijiazhuang 050024, China; 2 Key Laboratory of Optoelectronic Devices and Systems of Ministry of Education and Guangdong Province, Shenzhen University, Shenzhen, 518060, China; 3 Shenzhen key laboratory of laser engineering, College of Optoelectronic Engineering, Shenzhen University, Shenzhen, 518060, China

## Abstract

Topological insulators (TIs), are novel two-dimension materials, which can act as effective saturable absorbers (SAs) in a fiber laser. Moreover, based on the evanescent wave interaction, deposition of the TI on microfiber would create an effective SA, which has combined advantages from the strong nonlinear optical response in TI material together with the sufficiently-long-range interaction length in fiber taper. By using this type of TI SA, various scalar solitons have been obtained in fiber lasers. However, a single mode fiber always exhibits birefringence, and hence can support two orthogonal degenerate modes. Here we investigate experimentally the vector characters of a TI SA fiber laser. Using the saturated absorption and the high nonlinearity of the TI SA, a rich variety of dynamic states, including polarization-locked dark pulses and their harmonic mode locked counterparts, polarization-locked noise-like pulses and their harmonic mode locked counterparts, incoherently coupled polarization domain wall pulses, including bright square pulses, bright-dark pulse pairs, dark pulses and bright square pulse-dark pulse pairs are all observed with different pump powers and polarization states.

Ultrafast pulses have been widely used in material processing, optical communications, medicine, and optical sensing^1^,^2^,^3^,^4^. Passively mode-locked fiber lasers have also been demonstrated to be an excellent method for generating ultrafast pulses. In order to realize mode locked pulses, a saturable absorber (SA) is usually placed in the resonant cavity. In general, lasers that use nonlinear polarization rotation (NPR) as SAs are unstable. While laser based on nonlinear optical loop mirrors (NOLMs) or nonlinear amplifying loop mirrors (NALMs) with entirely polarization-maintaining cavity were demonstrated to be self-starting and stable^5^. Apart from NOLM or NALM, a material-based SA can also be used to generate stable mode-locked pulses. One type of material-based SA, the semiconductor saturable absorber mirror, however, always has a narrow transmission bandwidth and requires complex fabrication and packaging^6^,^7^. Another material-based SA, based on single-walled carbon nanotubes (SWCNTs) though easier to fabricate and cost effective, has working wavelengths that are connected with the diameter of the nanotubes^8^. A third material-based SA, graphene, has a higher optical damage threshold, lower loss and better wavelength-independence than SWCNTs. However, monolayer graphene always has a small absorption at 1550 nm^9^,^10^,^11^. Recently, several new and intensively investigated nanomaterials, including transition metal dichalcogenides (TMDCs), black phosphorus (BP) and topological insulators (TIs), have been used as SAs. However, TMDCs have sizeable bandgaps corresponding to only the visible and near-infrared spectrum ranges^12^, while BP is very sensitive to the environment because of the high reactivity of BP with air, and this might limit their applications in real devices^13^. Compared with above two nanomaterials, TIs have been found to have narrow topologically non-trivial energy gaps, and corresponding to a broadband saturable absorption. They have also been found to have a low saturable optical intensity, high damage threshold and large modulation depth^14^,^15^. Since Bernard *et al.* first demonstrated a TI-based SA in 2012, passive mode-locking or Q-switching operation has been achieved experimentally in different wavebands^16^,^17^,^18^,^19^,^20^. In addition, TIs also possess giant third order nonlinearity and have attracted much attention for their ability to generate short and high-energy laser pulses^17^,^21^. The TI nano-materials can composite with polymer to form thin films, and then were transferred to fiber end face to act as a SA^22^,^23^. Unfortunately, thermal effects can damage the fiber end surface as well as the TI SA. In order to overcome the above drawbacks, two types of TI SAs, made by depositing the TI directly onto the microfiber or side-polished fiber (D-shaped fiber) have been proposed^24^,^25^. Since only a part of optical intensity interacts with the TI in the cavity, these types of TI SAs can alleviate the thermal load on the fiber. In addition, the controllable length of the deposited material helps to increase the interaction length between the light and the TI. Note however, that compared with the microfiber, the insertion loss of the D-shaped fiber itself will increase dramatically if the single mode fiber (SMF) is polished to the core^26^.

By using a microfiber-based TI SA, harmonic mode-locked pulses, stable Q-switched pulses and multi-soliton pulses have all be obtained in fiber lasers^24^,^27^,^28^. However, in all these cases, only scalar solitons were obtained.

Since there exists asymmetries in the fiber waveguide caused during manufacturing, externally applied stress, or bending, a SMF always exhibits random birefringence, and hence can support two degenerate modes that are polarized in orthogonal directions. If the laser cavity does not include a polarizer, cross coupling between these two modes occurs, which results in different polarization dynamic states dependent on the different propagation velocities along the fiber. These states include group velocity locked polarization domains (PDs), and polarization locked (PL) pulses. Zakharov and Mikhailov first theoretically predicted the formation of PDs in nonlinear optics^29^. Malomed theoretically studied the polarization domain wall (PDW) between traveling waves by solving the coupled Ginzburg-Landau equations, and pointed out that PDWs might be realized as a boundary produced in two dimensions by a collision of waves traveling in different directions^30^. This prediction was confirmed experimentally by Pitois *et al.*^31^. Wabnitz and Daino theoretically studied the possibility of generating PD solitary waves in nonlinear optical fibers^32^, and Haelterman and Sheppard showed theoretically the existence of polarization domain wall soliton (PDWS) in a dispersive Kerr medium^33^,^34^. Williams and Roy observed PDs in a unidirectional erbium-doped fiber ring laser, and obtained square-wave pulses and irregular temporal patterns as operating parameters were changed^35^,^36^. Lecaplain presented a simple theoretical model to explain the different PDW complexes formed in fiber ring lasers operating with either a normal path-averaged dispersion or an average anomalous dispersion^37^,^38^. Tang *et al.* observed polarization resolved operation of a quasi-isotropic cavity, erbium-doped fiber laser and demonstrated the formation of PD^39^. Subsequently, Zhang *et al.* observed experimentally two types of phase-locked vector solitons in erbium-doped fiber lasers with weakly birefringent cavities^40^,^41^. Tang’s group experimentally observed soliton-dark pulse pair formation in a birefringent cavity fiber laser^42^. Recently, our group obtained group velocity locked PDs in an Yb-doped fiber laser with a quasi-square PD along one axis but with a chaotic-state PD along the other in the time domain^43^. A common feature of the above lasers was that they had no mode locker in the cavity. On the other hand, dark solitons in a fiber laser based on an NPR SA, or carbon nanotube SA have been observed^44^,^45^.

As mentioned above, a TI is a new type of SA, and a microfiber-based TI SA could effectively increase the interaction length between the light and the TI. Therefore, the question arises as to whether lasers with a microfiber-based TI SA can emit different PDs, or dark pulses. This was the initial motivation for our work.

In this paper, we experimentally investigated a net normal dispersion vector Er-doped fiber laser using a microfiber-based TI as the SA. This kind of SA can increase the length over which the light and the TI interact, and possess high nonlinearity. By adjusting the pump power and the PCs, various PDs with different widths and shapes, PL dark pulses and their harmonic mode locked counterparts, PL noise-like pulses and their harmonic mode locked counterparts could all be obtained.

## Results

### Experimental set-up and sample characterization

**Figure 1:**
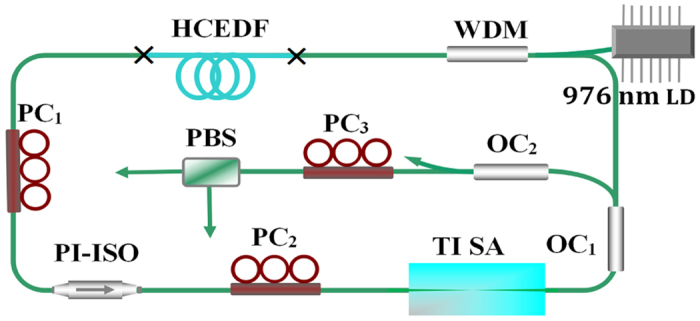
Schematic of Er-doped vector fiber laser. WDM: wavelength-division multiplexer; HCEDF: high concentration erbium-doped fiber; PC: polarization controller; PI-ISO: polarization-independent isolator; TI SA: topological insulator saturable absorber; OC_1_: 90:10 optical coupler; OC_2_: 50:50 optical coupler; PBS: polarization beam splitter.

A schematic diagram of the experimental set-up is shown in Fig. 1. The laser cavity contained a 4.6-m long high concentration erbium-doped fiber (HCEDF) with a group velocity dispersion (GVD) of 66.3 ps^2^ km^−1^ at 1550 nm, and a 13-m SMF with a GVD of −22 ps^2^ km^−1^. The net cavity dispersion was 0.019 ps^2^. A 976-nm laser diode was used to pump the HCEDF through a 980/1550 nm wavelength-division multiplexer (WDM). Two intra-cavity polarization controller (PC_1_) and PC_2_ were used to change the polarization state of the cavity. A polarization independent isolator (PI-ISO), whose polarization dependent loss was lower than 0.2 dB, was used to ensure the unidirectional operation of the ring cavity. A 90:10 output coupler (OC_1_) was used to output 10% of the cavity light. Passive mode locking was realized by using a microfiber-based TI SA. To observe the vector characteristics of the pulses, another extra-cavity PC_3_ and a fiber-based polarization beam splitter (PBS) were connected to OC_2_. An optical spectrum analyzer (Yokogawa AQ6317C) with a maximum resolution of 0.01 nm, a 1 GHz real-time oscilloscope (Yokogawa DL9140) with three 3-GHz photo-detectors and a commercial optical autocorrelator (FP-103XL) were used to observe the optical spectrum, temporal domain shape and pulse width.

In experiment, the TI was made of bismuth telluride (Bi_2_Te_3_), which was prepared as follows: Bi_2_Te_3_ bulk crystals were placed into a autoclave filled with an ethylene glycol solution of lithium hydroxide. The autoclave was then oven-heated to achieve intercalation of Bi_2_Te_3_ by the lithium ions dissolved in the solution. The dispersions in the solution were collected by filtration and rinsed with acetone. Colloidal suspensions of Bi_2_Te_3_ could be readily prepared by exfoliating the lithiated powder in deionized water. By filtering through porous polyvinylidene fluoride membranes, Bi_2_Te_3_ nanosheet membranes were obtained after drying^18^,^46^.

**Figure 2:**
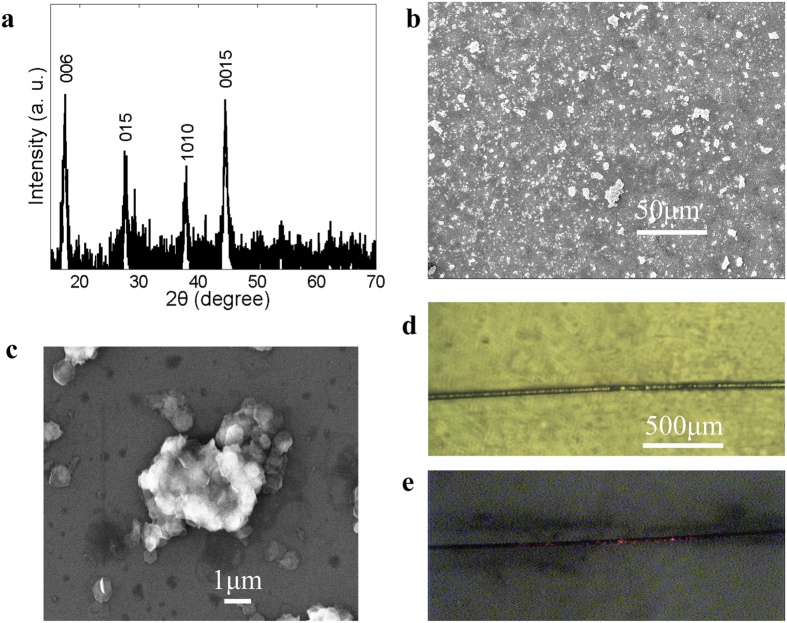
Characterization and measurement. (**a**) Measured XRD pattern of Bi_2_Te_3_. (**b**) Low-magnification SEM image of Bi_2_Te_3_ nanosheets. (**c**) High-magnification SEM image of Bi_2_Te_3_ nanosheets. (**d**) Microscope image of microfiber-based TI SA. (**e**) Microscope image of the evanescent field of the microfiber-based TI observed using visible light.

In order to characterize the TI, we measured the X-ray diffraction (XRD) pattern of the prepared Bi_2_Te_3_ nanosheets using a X-ray diffractometer (X’pert PRO MPD), as shown in Fig. 2a. Several distinct diffraction peaks, which correspond to the [006], [015], [1010], and [0015] crystal planes (JCPDS NO. 15-0863) can be indexed to Bi_2_Te_3_ (space group: R-3m) with lattice constants a = b = 0.438 nm, and c = 3.05 nm. In order to check the morphology of the Bi_2_Te_3_, we blended the powder with an ethanol solution and then ultrasonically processed it for half an hour at a power of 100 W. The suspension produced was then deposited on a silicon wafer using a pipette, and dried in vacuum. The resulting sample was observed using a scanning electron microscope (SEM). The lower magnification SEM image in Fig. 2b shows that the nanosheets were randomly dispersed on the silicon wafer.

A higher magnification SEM image shown in Fig. 2c indicates that the Bi_2_Te_3_ had a clear hexagonal structure. An aqueous suspension of Bi_2_Te_3_ produced as described above was also deposited on the surface of the microfibers constituting the SA. Figure 2d shows a highly enlarged microscope image of the fabricated microfiber-based TI SA measured by a ternary inverted metallurgical microscope (Jiangnan MR5000). The microscope image of the fabricated microfiber shown in Fig. 2d was used to measure the tapered fiber. The tapered fiber had a waist diameter of 30 μm and a core diameter of 2.16 μm. Assuming the refractive index of the core n_co_ and cladding n_cl_ were 1.454 and 1.45 respectively, one can calculate that the normalized frequency, V, was about 0.5 by using the equation:


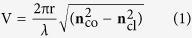


where r is the core radius, and λ = 1.56 μm is the wavelength. As Bilodeau pointed out, for a tapered fiber, when V is smaller than 0.84, the fundamental LP_01_ mode is no longer confined to the core but instead is guided by the cladding-air interface, which results in a mode field with the same diameter as the tapered fiber^47^,^48^,^49^. Consequently, we can deduce that the mode field diameter was also 30 μm in our experiment. We also observed the evanescent field of the microfiber-based TI by injecting visible light into the SA, as shown in Fig. 2e. The interaction between the evanescent field and the TI occurs only in an elongated area on one side of the optical fiber.

**Figure 3:**
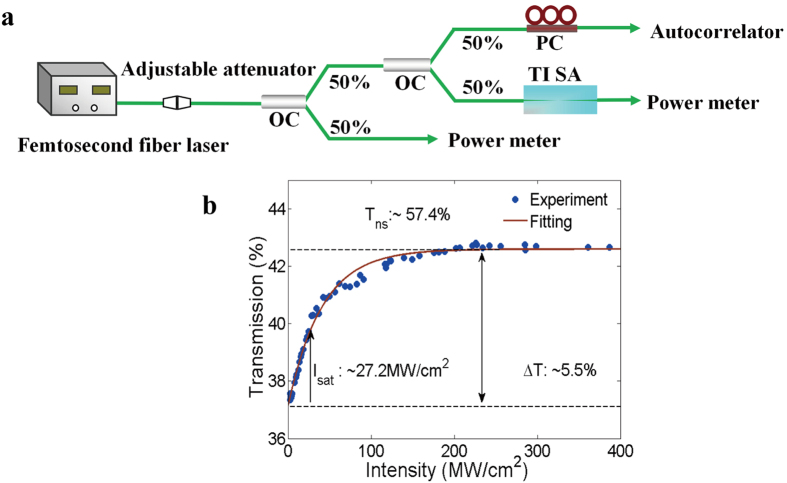
(**a**) Experimental setup for measurement of the nonlinear absorption of the microfiber-based TI; (**b**) measured transmission curve and the corresponding fitting curve.

To further investigate the characteristics of the TI, we measured its nonlinear absorption, using a femtosecond laser source with center wavelength of 1551.6 nm, repetition rate of 50 MHz, and a tunable pulse width (Calmar Opt-com FPL-04TTYSU11). The experimental setup is shown in Fig. 3a. Since the femtosecond pulse width decreased as the pump power increased, an optical autocorrelator was used to monitor the pulse width at the same time. Figure 3b shows the transmission curve of the microfiber-based TI transmission curve. By using the fitted equation of the nonlinear saturable absorption curve, T(I) = 1 − ∆T × exp(−I/I_sat_) − T_ns_, where T(I) is the transmission, ∆T is the modulation depth, I is the input peak power intensity, I_sat_ is the saturable intensity, and T_ns_ is the nonsaturable loss^50^, one can calculate that the modulation depth was 5.5%, the nonsaturable loss T_ns_ was 57.4%, and the saturable intensity was 27.2 MW/cm^2^.

In the experiment, we have also measured the polarization dependent loss (PDL) of the microfiber before and after deposing the TI. They were found to be 0.04 dB and 0.4 dB at 1550 nm, respectively. Since these PDLs value are small, we may conclude that the pulse operations described below were not caused by the NPR^51^.

## Experimental Observations

### Different interactions between the orthogonal linearly polarized eigenmodes

Since there were no polarizers in the cavity, the laser always simultaneously oscillated with two orthogonal linear polarization eigenmodes, and these two eigenmodes could interact along the fiber. By adjusting the intra-cavity PCs, the cavity birefringence could be altered and a rich set of PDW pulses could be obtained, including bright square pulses, bright-dark pulse pairs, uniformly distributed dark pulses, unevenly distributed dark pulses and bright square pulse-dark pulse pairs.

**Figure 4:**
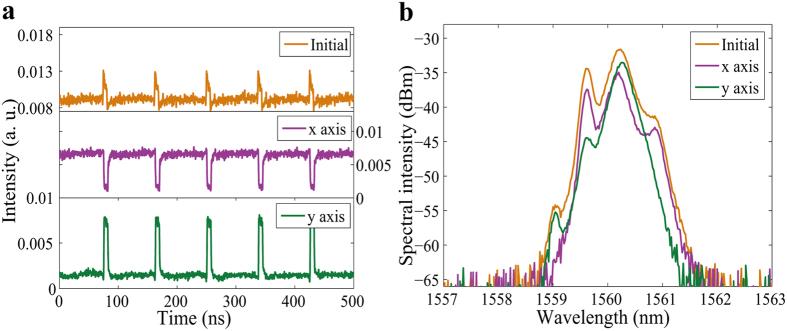
Bright square pulses. (**a**) Pulse traces before (Initial) and after (x axis and y axis) passing through the PBS; (**b**) optical spectra before (Initial) and after (x axis and y axis) passing through the PBS.

**Figure 5:**
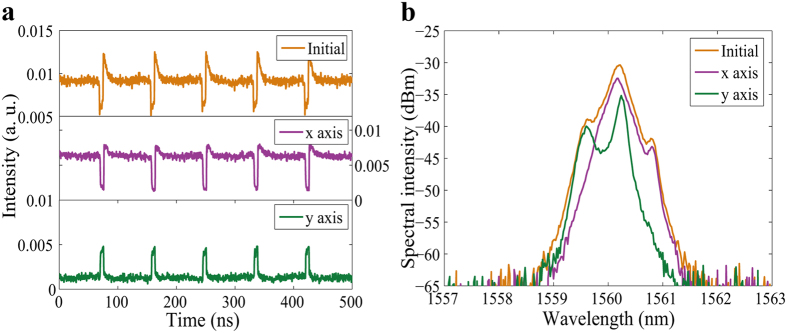
Bright-dark pulse pairs. (**a**) Pulse traces before (Initial) and after (x axis and y axis) passing through the PBS; (**b**) optical spectra before (Initial) and after (x axis and y axis) passing through the PBS.

When the pump power was increased to 255 mW and the intra-cavity PCs were carefully adjusted, two orthogonal modes oscillating simultaneously were easily formed. Though these orthogonal polarization components propagate with different group velocities in the fiber, they can trap one another through cross-phase modulation, thus enabling them to propagate as a single entity^39^,^52^. In addition, within one cavity round-trip period, the laser emission would switch from one polarization to the other, forming two PDs, which showed a wide width in x axis, while displayed a narrow width in y axis. As a result, a bright square pulse was formed in the total laser intensity output (Initial) of Fig. 4a. The spectra of the two linear polarization components (x axis and y axis) had different central wavelengths of 1560.02 nm and 1560.25 nm, as shown in Fig. 4b. Since the two PDs had a wavelength separation, the coupling was incoherent. Also, since there was only a small wavelength difference of 0.23 nm between the two orthogonal polarization components, the two orthogonal polarization components had only a small time delay as they propagated in the cavity. We may conclude that the net cavity birefringence was small in this state. Careful adjustment of the PCs away from the conditions for the bright square pulses, which corresponded to changing the net linear cavity birefringence, resulted in changing the widths and the shapes of the PDs. Figure 5 shows another manifestation of the PDs. The time domain graph shows that unlike the x axis of Fig. 4a, where the PDs have a square top, the PDs on the x axis of Fig. 5a have a slightly oblique top. Through incoherent coupling between the orthogonal linear polarization modes, the total emission shows a periodic bright-dark pulse pair, as shown in Fig. 5a (Initial). The corresponding polarization resolved spectra are shown in Fig. 5b. The 3-dB bandwidths were 0.32 nm for the x axis and 0.16 nm for the y axis and the corresponding central wavelengths were 1560.15 nm and 1560.23 nm, respectively. The separation of the wavelengths between the two parts was slight, which again indicates a small value of the average cavity birefringence.

**Figure 6:**
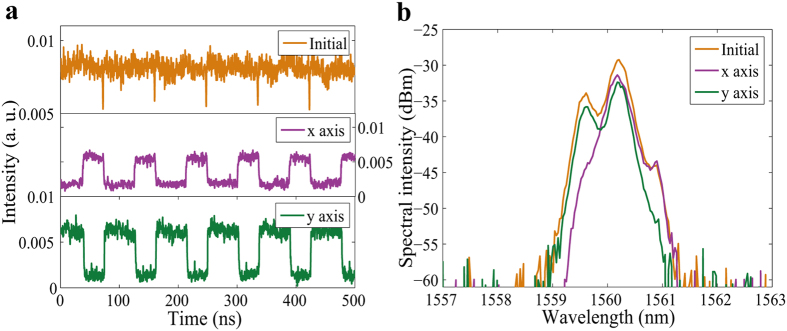
Fundamental dark pulses. (**a**) Pulse traces before (Initial) and after (x axis and y axis) passing through the PBS; (**b**) optical spectra before (Initial) and after (x axis and y axis) passing through the PBS.

When we decreased the pump power to 230 mW and adjusted PC_1_ and PC_2_, a dark pulse was observed before the PBS as shown in Fig. 6a. Since this dark pulse appeared at a polarization switching position of the total laser output intensity, and separated two PDs in a period, it was identified as a dark PDWS^34^. Figure 6a shows regularly distributed dark PDWS, for which the round trip time was 88 ns, which corresponds to the cavity length. It is obvious that the intensity of the pulses at the center of the dip did not fall to zero. These dark pulses are called “gray” pulses, and have been studied by our group, as reported in ref. 53. The central wavelengths of the two modes were 1560.16 nm and 1560.2 nm, and their spectral widths were 0.34 nm and 0.29 nm, as shown in Fig. 6b.

**Figure 7:**
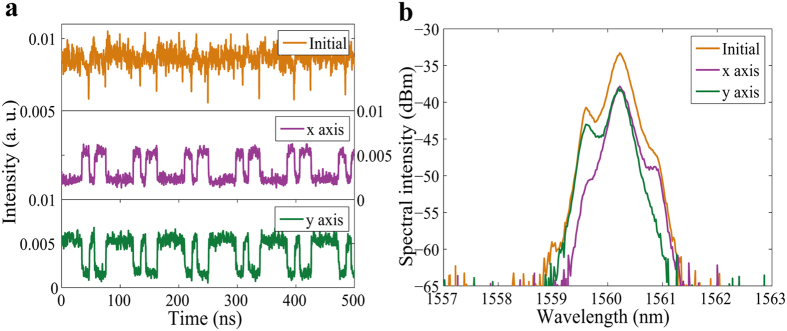
Unevenly distributed dark PDW pulses. (**a**) Pulse traces before (Initial) and after (x axis and y axis) passing through the PBS; (**b**) optical spectra before (Initial) and after (x axis and y axis) passing through the PBS.

When the pump power was slightly increased to 248 mW, splitting of the PDs occurred. As an example, Fig. 7a shows one of the split states, in which four PDs were formed in the cavity. Correspondingly, irregularly distributed PDWSs were also obtained (see Fig. 7a initial trace). That is to say, depending on the cavity parameters, the PDWSs could have different forms. In addition, the PDWSs had different depths in the time domain. The polarization resolved spectra are shown in Fig. 7b. We found that the separation of the central wavelengths of the two components was still small. The 3-dB bandwidths were 0.33 nm and 0.32 nm.

**Figure 8:**
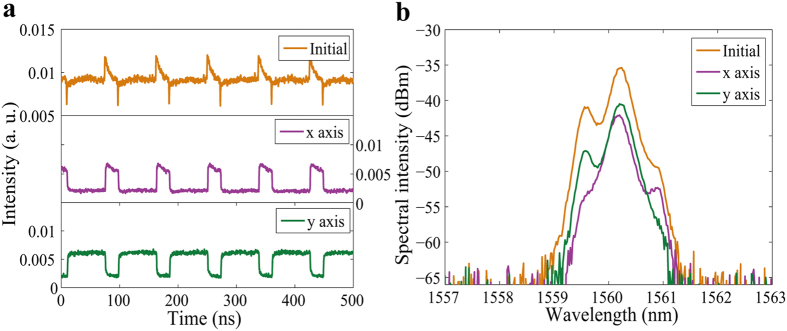
Bright square pulse-dark pulse pairs. (**a**) Pulse traces before (Initial) and after (x axis and y axis) passing through the PBS; (**b**) optical spectra before (Initial) and after (x axis and y axis) passing through the PBS.

Very interestingly, by further adjusting the intra-cavity PCs, another PDWS form consisting of bright square pulse-dark pulse pairs was also obtained in the total laser intensity output, as shown in Fig. 8a (Initial). The time domain graph shows that like the domain for the x axis of Fig. 5a, the PDs for the x axis of Fig. 8a also have a slightly oblique top. The total emission then showed a periodic bright square pulse-dark pulse pair. The spectra of the two modes on the x axis and y axis have slightly different central wavelengths of 1560.18 nm and 1560.22 nm, and the spectral widths are 0.36 nm and 0.35 nm, as shown in Fig. 8b.

### Polarization-locked vector dark pulses

Through increasing the pump power and carefully adjusting intra-cavity PCs, we obtained vector dark pulses. Further adjusting the PCs, which corresponded to tuning the wavelengths of the laser oscillations, the wavelength separation between the two orthogonal vector dark pulses could be tuned to zero. In this case, the group velocity difference could be assumed to be negligible. Then the orthogonal vector dark pulses maintained their temporal and polarization state profiles during propagation within the birefringent environment. Such dark pulses could be referred to as phase- or PL vector dark pulses^54^,^55^,^56^.

**Figure 9:**
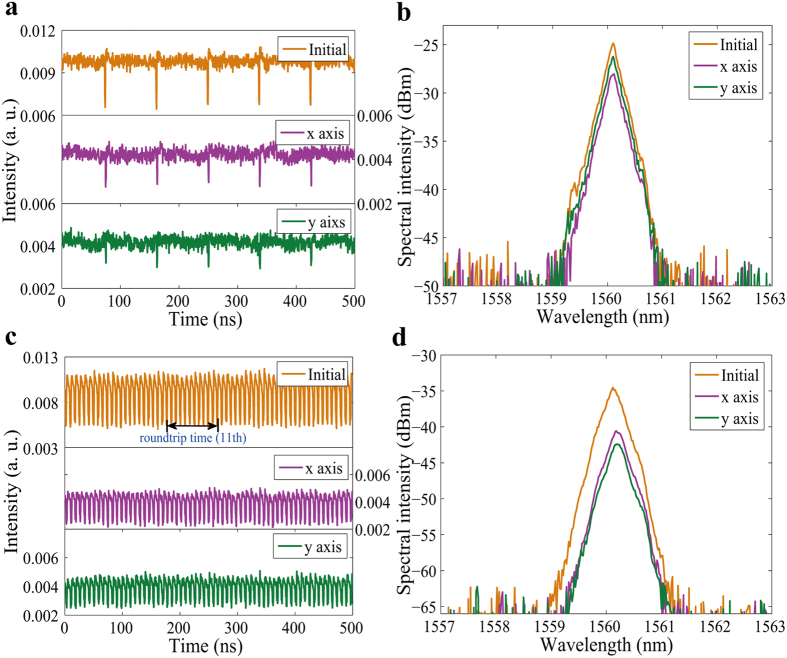
PL vector dark pulses. (**a**) Pulse traces of fundamental vector dark pulses before (Initial) and after (x axis and y axis) passing through the PBS and (**b**) corresponding optical spectra; (**c**) pulse traces of high order harmonic mode-locked vector dark pulses and (**d**) corresponding optical spectra.

With a pump power of 265 mW, we first obtained PL fundamental dark pulses as shown in Fig. 9a. The two components shared the same repetition rate of 11.4 MHz, which was consistent with the cavity length. Figure 9b shows that the two polarization components have same central wavelength of 1560.10 nm.

With a further increase in the pump power, we also obtained PL high order harmonic mode-locked (HML) dark pulses. In order to protect the laser pump source, the highest order we obtained was 11, which had a repetition rate of 125.4 MHz, as shown in Fig. 9c. The two polarization components had the same central wavelength of 1560.16 nm as shown in Fig. 9d. We also found that the spectral intensity difference between the two orthogonal polarization components was less than 3 dB. These observations further confirmed that they were PL HML dark pulses.

### Polarization-locked noise-like pulses

**Figure 10:**
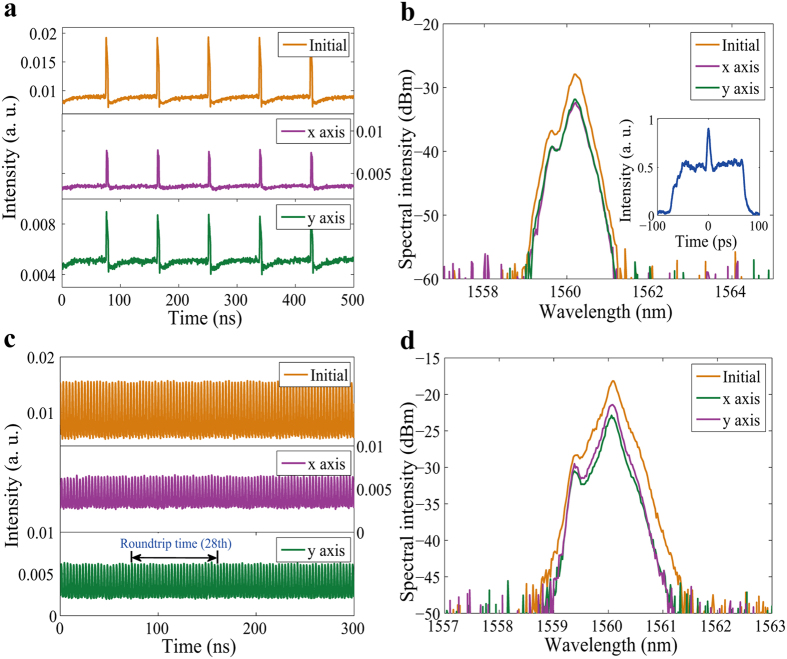
PL noise-like pulses. (**a**) Pulse traces of fundamental noise-like pulses before (Initial) and after (x axis and y axis) passing through the PBS and (**b**) corresponding optical spectra (Inset: autocorrelation trace of the total pulse); (**c**) pulse traces of high order harmonic mode-locked noise-like pulses and (**d**) corresponding optical spectra.

By further rotating the PCs while holding the pump power at 248 mW, we also obtained noise-like pulses, as shown in Fig. 10. The inset in Fig. 10b shows the autocorrelation trace of a single pulse before passing through the PBS, and we can see that there is a coherent peak riding on a wide shoulder that extends over the entire scanning time window, which indicates that a noise-like pulse was formed. We also found that the two parts had the same central wavelength of 1560.12 nm (see Fig. 10b) and that the pulse trains were uniform in spacing both before and after passing through the PBS (see Fig. 10a), which showed that they were PL fundamental noise-like pulses.

As the pump power was further increased to 270 mW, the fundamental noise-like pulses began to split, and high order HML noise-like pulses were formed. The highest order obtained was 28, as shown in Fig. 10c. The spectra in Fig. 10d shows that the two orthogonal polarization components had the same central wavelength of 1560.04 nm, which indicates that they were PL high order HML noise-like pulses.

It has previously been found experimentally that noise-like pulses are generated in a cavity with mode locking mechanisms such as NPR, SWCNTs, figure-eight, etc.^57^,^58^,^59^. Therefore, the observation of the PL noise-like pulses can be attributed to the microfiber-based TI SA.

## Discussion

In conclusion, we have demonstrated an Er-doped mode locked vector fiber laser that used a microfiber-based topological insulator as a saturable absorber. The experimental results showed that the formation of bright square pulses, bright-dark pulse pairs, uniformly distributed dark pulses, unevenly distributed dark pulses and bright square pulse-dark pulse pairs all resulted from the existence of PDs and that the high nonlinearity provided by the microfiber-base TI SA is favorable for the splitting of the PDs. Since neither the formation of the PDs nor the high nonlinearity are unique properties of the TI used in this work, we may infer that the different pulse generation regimes seen here are possible using other nanomaterial based SA with high nonlinearity. In fact, Gao *et al.* have demonstrated PDs in a mode-locked fiber laser based on reduced graphene oxide^60^. They found that reduced graphene oxide could provide both saturable absorption and high nonlinearity.

On the other hand, since the interaction between the evanescent field and the TI occurred only in an elongated area on one side of the optical fiber, we could obtain only PL noise-like pulses and their HML counterparts. If the microfiber-based topological insulator had perfect evanescent field properties, stable mode-locked pulses would be obtained.

## Additional Information

**How to cite this article**: Liu, J. *et al.* Polarization domain wall pulses in a microfiber-based topological insulator fiber laser. *Sci. Rep.* **6**, 29128; doi: 10.1038/srep29128 (2016).
